# Supplementary material for: Clathrin coats partially preassemble and subsequently bend during endocytosis
Source: J Cell Biol. 2023 Feb 3;222(3):e202206038. doi: 10.1083/jcb.202206038 (PMC9929656; doi:10.1083/jcb.202206038)
Supplement: Appendix — shows supplementary data also available in the BioStudies database (https://www.ebi.ac.uk/biostudies/) under accession number S-BIAD566. [file JCB_202206038_TextS1.pdf]

# Supplementary note to: Superresolution microscopy reveals partial preassembly and subsequent bending of the clathrin coat during endocytosis

Markus Mund, Aline Tschanz, Yu-Le Wu, Felix Frey, Johanna Mehl,
Marko Kaksonen, Ori Avinoam, Ulrich S. Schwarz, and Jonas Ries

## I. COOPERATIVE CURVATURE MODEL

We derive a kinetic model for clathrin-mediated endocytosis that is based on minimal assumptions based on our experimental observations. We first assume that the area $A$ of clathrin coats grows mainly by addition of new triskelia at the edges [1]. With a local growth rate $k_{\rm on}$ and an edge length $\mathcal{E}$, we have the simple growth law

$$\frac{\mathrm{d}}{\mathrm{d}t} A = \dot{A} = k_{\rm on}\mathcal{E} \,. \tag{1}$$

We also know from our experimental observations that all clathrin-coated pits have the geometry of a spherical cap with area $A_{\rm cap} = 2\pi R^2(1-\cos\theta)$, where $R$ is the cap radius and $\theta$ the closing angle. The edge length then is $\mathcal{E}_{\rm cap} = 2\pi R \sin\theta$. Using these formulas on Eq. (1) we find

$$2\dot{R}\tan\frac{\theta}{2} + R\dot{\theta} = k_{\rm on} \,. \tag{2}$$

Because we parametrize the shape of the growing clathrin coat by the two dynamical variables $R(t)$ and $\theta(t)$, but have only one growth equation up to now, we have to make additional assumptions to completely define our kinetic model.

As the main function of clathrin is to generate curvature, we now focus on coat curvature $H = 1/R$ and assume that it increases with a basal rate $\gamma$. Experimentally we observe that clathrin patches first grow flat and then start to curve. Curvature generation in clathrin lattices is the combination of a preferred curvature of the single triskelion and cooperative effects in the lattice [2, 3]. Several mechanisms exist that might generate curvature during coat growth. First, clathrin triskelia are likely to be geometrically frustrated within the flat clathrin coat. To overcome this frustration, they would have to adjust their positions relative to their neighbors, such that their free energy is getting more favorable. Second, lattice vacancies could be filled up with new triskelia, as predicted theoretically [4]. Note that these vacancies would not necessarily relate to patch area, because the clathrin lattice consists of many overlapping arms. Filling of vacancies would however increase clathrin density and coat stiffness, and thus could also drive invagination of the coat [5]. We note that it was indeed confirmed experimentally that the clathrin density increases within the coat during invagination [2, 6]. Third, lattice pentagons, associated with lattice curvature, could form at the edge of the clathrin lattice and diffuse into the coat, similar to lattice defects on curved surfaces [7]. All three mechanisms could generate curvature with a certain rate which we assume to be constant during the initial stages of growth.

At late stages, increase of curvature has to stop and therefore we assume curvature saturation at a characteristic value $H_0$, which is similar to, but different from the radius measured for clathrin cages. A simple estimate would be 40 nm for a typical radius of the membranes in the pits [8] plus 15 nm thickness of the clathrin coat [9]. These 55 nm would be larger than the 40 nm for clathrin cages, which are expected to be frustrated. If all triskelia in the lattice had achieved their optimal positions, a preferred or spontaneous curvature $H_0$ should emerge. Thus a growth equation for $H$ should have a stable fixed point generated by a higher order term. Since the corresponding mechanism is known to be of cooperative nature, larger groups of triskelia should be involved and the mechanism should start to dominate relatively late in the process, but then dominate quickly. Here we assume that the mechanism is proportional to $H^2$ (rather than to $H$, with a linear law corresponding to a non-cooperative effect that starts to show early). Assuming that curvature generation is a geometrical effect, its time development should depend on the closing angle and we get

$$\frac{\mathrm{d}H}{\mathrm{d}\theta} = \gamma\left(1 - \frac{H^2}{H_0^2}\right) \,. \tag{3}$$

The stable fixed point at $H = H_0$ can then be interpreted as corresponding to the preferred curvature of the mature coat.

Eq. (3) can be solved with the initial condition $H(\theta = 0) = 0$ by

$$H(\theta) = H_0 \tanh\left(\frac{\gamma}{H_0}\theta\right) \,, \tag{4}$$

TABLE 4. Coat curvature, coat area, and edge length summarized for the CCM, the CAM, and the CoopCM.

| | CCM | CAM | CoopCM |
|---|---|---|---|
| $H$ | $1/R_{\mathrm{CCM}}$ | $\sqrt{\frac{2\pi(1-\cos\theta)}{A_{\mathrm{CAM}}}}$ | $1/R_{\mathrm{coat}}$ |
| $A$ | $2\pi R_{\mathrm{CCM}}^2(1-\cos\theta)$ | $A_{\mathrm{CAM}}$ | $2\pi R_{\mathrm{coat}}^2(1-\cos\theta)$ |
| $\mathcal{E}$ | $2\pi R_{\mathrm{CCM}}\sin\theta$ | $\sqrt{\frac{2\pi A_{\mathrm{CAM}}}{(1-\cos\theta)}}\sin\theta$ | $2\pi R_{\mathrm{coat}}\sin\theta$ |

and defines the coat radius as a function of the closing angle

$$R_{\mathrm{coat}} = R(\theta) = \frac{1}{H(\theta)} = R_0 \frac{1}{\tanh(\gamma R_0 \theta)}\,, \tag{5}$$

with $R_0 = 1/H_0$. We note that the expansion of Eq. (5) gives $R \propto 1/\theta$ in leading order around the flat state.

We can combine Eq. (2) and Eq. (3) to find the full dynamics of coat invagination. Therefore, we compute the derivative of $R_{\mathrm{coat}}$

$$\frac{\mathrm{d}R_{\mathrm{coat}}}{\mathrm{d}\theta} = -\frac{\gamma R_0^2}{\sinh^2(\gamma R_0 \theta)}\,. \tag{6}$$

We use Eq. (5) and Eq. (6) and the chain rule $\mathrm{d}R/\mathrm{d}t = \mathrm{d}R/\mathrm{d}\theta\,\mathrm{d}\theta/\mathrm{d}t$ to rewrite Eq. (2). Solving for $\dot{\theta}$ yields

$$\dot{\theta} = k_{\mathrm{on}} \frac{1}{2\frac{\mathrm{d}R(\theta)}{\mathrm{d}\theta}\tan\frac{\theta}{2} + R(\theta)}\,. \tag{7}$$

After expanding Eq. (7) up to leading order in $\theta$ we find

$$\dot{\theta} = \frac{12\gamma k_{\mathrm{on}}}{8\gamma^2 R_0^2 - 1}\frac{1}{\theta}\,, \tag{8}$$

which is solved with the initial condition $\theta(t=0) = 0$ by

$$\theta(t) = \sqrt{\frac{24\gamma k_{\mathrm{on}}}{8\gamma^2 R_0^2 - 1}t}\,. \tag{9}$$

Thus the coat initially is flat ($\theta = 0$) and then starts to generate curvature, increasing the closing angle with a square root dependence in time, which reflects the slowing down of curvature generation as preferred curvature is approached. We refer to this model as the cooperative curvature model (CoopCM) due to the assumption of a non-linear growth law.

## II. FIT RESULTS

To contrast the CoopCM with existing models, we fit it to experimental data for $H(\theta)$, $A(\theta)$ and $\mathcal{E}(\theta)$. We compare the fitted curves to the constant curvature model (CCM), defined through the constant coat curvature $1/R_{\mathrm{CCM}}$, and to the constant area model (CAM), defined through the constant coat area $A_{\mathrm{CAM}}$. The equations for the curvature $H(\theta) = 1/R(\theta)$, the area $A(\theta)$, and the edge length $\mathcal{E}(\theta)$ are summarized in Table 4 for all three models. In order to fit, we first filter the data according to curvature $H$, as described in the methods section of the main text. We then fit the different models to each of the data sets separately. For the CCM and CAM we get values for one parameter each, namely $R_{\mathrm{CCM}}$ and $A_{\mathrm{CAM}}$, respectively. For the CoopCM we obtain two parameter values $\gamma$ and $H_0$. For the CoopCM we also determine the area when invagination occurs $A_0 = A(\theta = 0.01)/A(\theta = \pi)$, which is the relative transition size where the flat-to-curved-transition occurs. By definition, invagination in the CCM starts simultaneously with coat assembly ($A_0 = 0$), while invagination in the CAM starts at the maximum area ($A_0 = 1$). Moreover, for the CoopCM we also compute $R_0$. For all parameters we also determine the relative fit errors, given by $\sigma_x/x$, where $\sigma_x$ is the standard deviation of the parameter $x$ and $x$ is the value of the parameter. This procedure allows us to compare the fit results to each other. The fitted parameter values for the different models and cell lines are summarized in Table 1–3.

We then fit Eq. (9) to the data, describing $\theta(t)$. Using the values of $\gamma$ and $H_0$, we determine $k_{\mathrm{on}}$ from the fit. The resulting values, measured in units of the pseudo time $\tilde{s}$, are summarized in Table 1–3. We do not show the fit errors of $k_{\mathrm{on}}$, because they are small and result from the same fit of $\theta(t)$ and the errors of $\gamma$ and $H_0$, which does not allow us to discriminate between the different fits.

TABLE 5. BIC for the curvature fits $H(\theta)$ of the CCM, CAM and CoopCM for the analyzed cell lines.

| | CCM | CAM | CoopCM |
|---|---|---|---|
| SK-MEL-2 cells | -19079 | -21218 | -21670 |
| c3T3 cells | -8119 | -9179 | -9498 |
| U2OS cells | -2784 | -3097 | -3190 |

## III. BAYESIAN INFORMATION CRITERION

Apparently, the CoopCM agrees better with the data compared to the CCM and the CAM. However, while the CCM and the CAM both only include one parameter, the CoopCM has two parameters. In order to base model selection on a statistical criterion and to account for the additional parameter of the CoopCM, we compute the Bayesian information criterion (BIC). The BIC takes into account both how well a model fits the data but also how many free parameters it includes to avoid overfitting. The BIC can be expressed by [10]

$$\mathrm{BIC} = N \ln \left( \frac{\mathrm{RSS}}{N-k} \right) + k \ln \mathrm{N} \approx N \ln \left( \frac{\mathrm{RSS}}{N} \right) + k \ln \mathrm{N} \,, \tag{10}$$

where we used that $N \gg k$ in the last step. Here RSS is the residual sum of squares $RSS = \Sigma_i^N (f(x_i) - y_i)^2$ with $x_i$ and $y_i$ the measured data and $f(x_i)$ the fitted data, $N$ is the number of data points and $k$ is the number of free parameters. In general, when comparing different models, the model with the lowest BIC is preferred. In Table 5 the BIC is calculated from Eq. (10) for the curvature fits $H(\theta)$ for the different cell lines. For all cell lines, the BIC for the CoopCM is lowest. Therefore, we conclude that the additional parameter of the CoopCM is justified and that the CoopCM agrees best with the data.

## IV. LINEAR CURVATURE MODEL

As an alternative to the CoopCM model we now consider a linear curvature model. As in the CoopCM model, we assume that the coat curvature $H = 1/R$ increases at a basal rate $\gamma$. In contrast to the CoopCM model however, we now assume that the increase in curvature at late stages is slowed down proportional to $H$ and must stop at a value $H_0$

$$\frac{\mathrm{d}H}{\mathrm{d}\theta} = \gamma \left( 1 - \frac{H}{H_0} \right) . \tag{11}$$

Eq. (11) can be solved by

$$H(\theta) = H_0 \left( 1 - \mathrm{e}^{-\frac{\gamma}{H_0}\theta} \right) . \tag{12}$$

Eq. (12) also defines the coat radius $R = 1/H$ as a function of the invagination angle $\theta$

$$R(\theta) = R_0 \frac{1}{(1 - \mathrm{e}^{-\gamma R_0 \theta})} \,, \tag{13}$$

with $R_0 = 1/H_0$. The derivative of $R(\theta)$ reads

$$\frac{\mathrm{d}R}{\mathrm{d}\theta} = - \frac{\gamma R_0^2}{4 \left( \sinh \frac{\gamma R_0 \theta}{2} \right)^2} . \tag{14}$$

Now we use Eq. (13) and Eq. (14) on Eq. (7). After expanding up to second order in $\theta$ we find

$$\dot{\theta} = \frac{2k_{\mathrm{on}}}{R_0} \left( 1 - \frac{1}{6} \left( 2\gamma R_0 - \frac{1}{\gamma R_0} \right) \theta \right) \,, \tag{15}$$

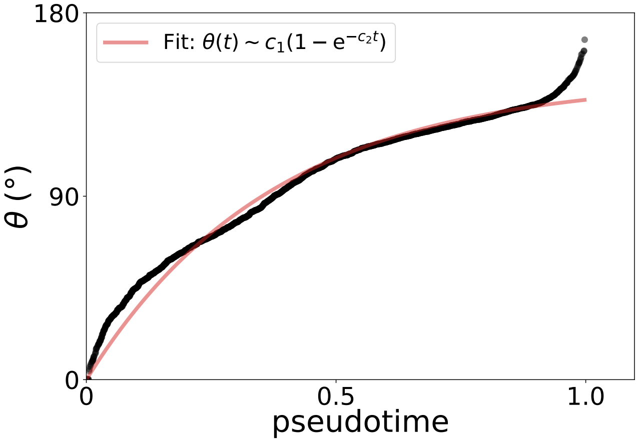


Supplementary Figure 6. Invagination angle $\theta$ as a function of the pseudotime $t$. Red line: the fit to the same data as in Fig. 4B according to Eq. (16) for the linear curvature model.

which is solved by

$$\theta(t) = \frac{1}{R_0}\left(\frac{3\gamma}{2\gamma^2 - \frac{1}{R_0^2}}\right)\left(1 - \mathrm{e}^{-\left(\frac{2\gamma^2 - \frac{1}{R_0^2}}{3\gamma}\right)k_{\mathrm{on}}t}\right). \tag{16}$$

For small t, Eq. (16) can be expanded to $\theta(t) \sim (k_{\mathrm{on}}/R_0)t$. The linear increase of $\theta$ with t does not fit the data for small $t$ (cf. Supplementary Figure 6).


[1] D. Bucher, F. Frey, K. A. Sochacki, S. Kummer, J.-P. Bergeest, W. J. Godinez, H.-G. Kräusslich, K. Rohr, J. W. Taraska, U. S. Schwarz, *et al.*, Clathrin-adaptor ratio and membrane tension regulate the flat-to-curved transition of the clathrin coat during endocytosis, Nat. Commun. **9**, 1109 (2018).

[2] W. F. Zeno, J. B. Hochfelder, A. S. Thatte, L. Wang, A. K. Gadok, C. C. Hayden, E. M. Lafer, and J. C. Stachowiak, Clathrin senses membrane curvature, Biophys. J. , S0006349521000801 (2021).

[3] K. A. Sochacki, B. L. Heine, G. J. Haber, J. R. Jimah, B. Prasai, M. A. Alfonzo-Mendez, A. D. Roberts, A. Somasundaram, J. E. Hinshaw, and J. W. Taraska, The structure and spontaneous curvature of clathrin lattices at the plasma membrane, bioRxiv 10.1101/2020.07.18.207258 (2020).

[4] F. Frey, D. Bucher, K. A. Sochacki, J. W. Taraska, S. Boulant, and U. S. Schwarz, Eden growth models for flat clathrin lattices with vacancies, New J. Phys. **22**, 073043 (2020).

[5] F. Frey and U. S. Schwarz, Competing pathways for the invagination of clathrin-coated membranes, Soft Matter **16**, 10723 (2020).

[6] K. A. Sochacki, A. M. Dickey, M.-P. Strub, and J. W. Taraska, Endocytic proteins are partitioned at the edge of the clathrin lattice in mammalian cells, Nat. Cell Biol. **19**, 352–361 (2017).

[7] S. Agarwal and S. Hilgenfeldt, Simple, General Criterion for Onset of Disclination Disorder on Curved Surfaces, Phys. Rev. Lett. **125**, 078003 (2020).

[8] O. Avinoam, M. Schorb, C. J. Beese, J. A. Briggs, and M. Kaksonen, Endocytic sites mature by continuous bending and remodeling of the clathrin coat, Science **348**, 1369 (2015).

[9] M. Saleem, S. Morlot, A. Hohendahl, J. Manzi, M. Lenz, and A. Roux, A balance between membrane elasticity and polymerization energy sets the shape of spherical clathrin coats, Nat. Commun. **6**, 6249 (2015).

[10] R. A. Yaffee and M. McGee, *An introduction to time series analysis and forecasting: with applications of SAS® and SPSS®* (Elsevier, 2000).
